# Identification of the *Chenopodium quinoa HSP90* Gene Family and Functional Analysis of *CqHSP90.1c* and *CqHSP90.6a* Under High-Temperature Stress in Transgenic *Arabidopsis thaliana*

**DOI:** 10.3390/plants14172770

**Published:** 2025-09-04

**Authors:** Fangjun Chen, Wei Wang, Wenli Liu, Jiancheng Song, Shihua Chen, Yibo An, Haibo Yin, Shanli Guo

**Affiliations:** 1 College of Life Sciences, Yantai University, Yantai 264005, China; 2 Shandong Laboratory of Advanced Agriculture Sciences at Weifang, Peking University Institute of Advanced Agricultural Sciences, Weifang 261325, China; 3 Chongqing Forestry Investment Development Co., Ltd., Chongqing 401120, China

**Keywords:** quinoa, *HSP90*, expression pattern, gene family, bioinformatics analysis

## Abstract

Heat shock protein 90 (HSP90) is an evolutionarily conserved molecular chaperone. Numerous studies have shown that it is widely involved in protein folding, assembly, stabilization, activation, and degradation in response to various biotic and abiotic stresses in many normal cellular processes and under stress conditions. We identified 11 members of the *CqHSP90* gene family in the quinoa (*Chenopodium quinoa*) genome by bioinformatics analysis. Phylogenetic tree analysis showed that quinoa was more closely related to dicotyledonous plants than to monocotyledonous plants. Quinoa is susceptible to high-temperature stress during its growth and development. We analyzed the cis-acting elements of its promoter, and found that nearly 1/3 of the cis-acting elements were stress-responsive, and 2/3 of them had heat-responsive elements. The results of qRT-PCR showed that heat shock at 40 °C could induce a high expression of *CqHSP90.1c* and *CqHSP90.6a*. Subcellular localization indicates that they are all membrane proteins. At the same time, both *CqHSP90.1c* and *CqHSP90.6a* overexpression lines improved the tolerance of *Arabidopsis thaliana* under high temperature, indicating that both of them had a positive regulatory effect under heat stress. Results of this study could provide useful information for further study on the biological role of *CqHSP90.1c* and *CqHSP90.6a*, and provide theoretical basis for quinoa resistance breeding.

## 1. Introduction

Plants are inevitably exposed to abiotic primary stresses such as high light intensity, thermal shock, heavy metals, salinity, drought and low temperature, and secondary stresses such as osmosis and oxidation [1,2]. This has caused some adverse effects on plant growth and development, but in the process of evolution, plants have also widely acquired stress response factors, through which some mechanisms of action can be produced to adapt morphologically and physiologically. The biosynthesis of many proteins called “stress proteins” is induced to protect the organism from these harmful stimuli [3,4]. It is well known that the function of any protein is determined by the formation and folding into a three-dimensional structure [5]. Potentially, most stressors that induce protein misfolding require HSP/chaperone recruitment. HSP90 is involved in the regulation of a variety of proteins and the maintenance of normal protein configuration, and is widely involved in various cellular stress resistance activities [6]. The wrong stacking of proteins can lead to many problems in cells, so organisms have formed highly specific mechanisms to maintain protein balance in vivo and alleviate the impact of adversity on organisms [7]. In this regard, HSP partners are considered a powerful buffer against environmental stress and even genetic variation [8].

HSP90 is part of the Gyrase, HSP90, and Histidine Kinase and MutL (GHKL) superfamily of ATPases [9]. HSP90 proteins are widely distributed in the cytoplasm, chloroplasts, mitochondria, and endoplasmic reticulum, and their structures are highly conserved, consisting of three domains, namely, a highly conserved ATP-binding domain located at the N-terminus, a middle domain, and a dimerization domain located at the C-terminus [6]. Based on molecular weight and sequence homology, HSPs can be divided into five protein families: HSP100S/ClpB, HSP90S, HSP70S/DnaK, HSP60S, and HSP20S. HSP90 represents about 1% to 2% of the total eukaryotic proteins [10]. The molecular mechanism of heat shock protein function has been widely demonstrated [11,12,13]. Among them, HSP70 and HSP90 and their co-chaperones have been clearly demonstrated to be associated with signaling and protein-targeted degradation [14,15,16]. HSP90 plays an important role in normal cell development, such as the formation of spatial structure of kinase substrates, initial stress signal transduction, and the maintenance of spatial structure of transcription factors [17,18,19].

Heat shock protein 90 has important biological functions in both animals and plants. In yeast and vertebrates, HSP90 is abundantly expressed in the cytoplasm at room temperature and is rapidly accumulated in the nucleus under heat shock condition [20]. Proteomic and phosphoproteomic analyses of plants in recent years have shown that many HSP90 proteins exist under drought and salt stress, and may be involved in signal transduction during stress response [21,22,23]. *AtHSP90* has been widely studied to play an important role in abiotic stress resistance, plant hormone response and light-dark signal conversion [24,25,26]. Meanwhile, *AtHSP90.5* was shown to play an important role in chloroplast biogenesis and embryogenesis [27,28]. Studies in cotton (*Gossypium hirsutum*) have shown that HSP90 plays a crucial role in maintaining cellular homeostasis and in cotton fiber differentiation and development [29]. In tobacco (*Nicotiana tabacum*), studies have shown that HSP90 also has some resistance to biological stress, and HSP90 interacts with Required for Mla12 Resistance (RAR1) and Toll/interleukin-1 receptor–nucleotide-binding leucine-rich repeat (TIR-NB-LRR) to improve the resistance of leaves to mosaic virus [30]. In conclusion, HSP90 plays an important role in plant growth and development, stress response, and resistance to pests and diseases. Therefore, further study on the function of *HSP90* in plants is helpful to understand stress signal transduction, key stress-related gene discovery, and crop resistance improvements. However, the *HSP90* gene family of quinoa, which is an excellent crop with strong resistance and adaptability to extreme environments, has not yet been identified and analyzed. Therefore, it is of great significance to further study the function of HSP90 in plant stress and to analyze the stress signal transduction.

In this study, quinoa *HSP90* genes were identified using bioinformatics methods. The expression patterns of different tissue structures of the *CqHSP90* gene family were analyzed using the existing RNA-seq data (PRJNA394651). The expression patterns of the *HSP90* gene family under high temperature were determined using qRT-PCR. At the same time, the three-dimensional structure of the protein and its protein–protein interaction (PPI) network were predicted to reveal the possible regulatory mechanism of HSP90s. The results of this study will provide valuable information for further study of the function and regulation mechanism of the *HSP90* gene in the regulation of abiotic stress in quinoa.

## 2. Results

### 2.1. Identification and Physicochemical Properties of the CqHSP90 Gene Family

Based on quinoa genome-wide information relating to HATPase_C (PF02518) and HSP90 domains (PF00183), a total of 11 *HSP90* gene family members were identified (Table S1, Figure S1) and named according to their evolutionary relationship with *Arabidopsis thaliana*. As shown in Table 1, the HSP90 encoding protein was consistent with the definition of HSP90 protein, with an amino acid length of 697–810 aa and molecular weight of 79.9–93.1 kDa. This indicated that there is no loss of genetic sequences in the process of evolution. The isoelectric points (pI) of all HSP90-encoded proteins ranged from 4.83 to 5.3, indicating that all CqHSP90 proteins were acidic (pI < 7). CqHSP90.4a, CqHSP90.4b, CqHSP90.7a, and CqHSP90.7b also have similar molecular weight, isoelectric point, and subcellular localization. CqHSP90.1c and CqHSP90.1b also have the same molecular weight and isoelectric point. Meanwhile, their subcellular localizations are different. The results of subcellular localization showed that HSP90 was mainly distributed in nucleus (Nucl), mitochondria (Mito), endoplasmic reticulum (E.R.), chloroplast (Chlo), and cytoplasm (Cyto). This means that they may play important roles in stress tolerance or development in specific organelles.

### 2.2. Phylogenetic Analysis of the HSP90 Gene Family

**Figure 1:**
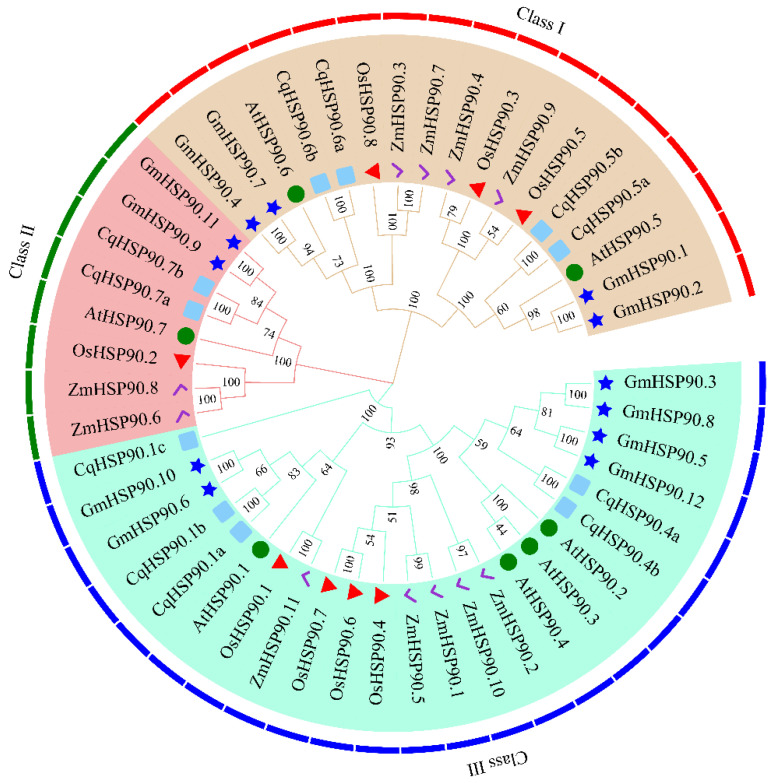
Phylogenetic analysis of HSP90 proteins in quinoa, soybean, maize, Arabidopsis, and rice. The phylogenetic tree of HSP90 protein was constructed using MEGA-7 software. The three classes are represented by different colors. Red triangles represent rice HSP90s (OSHSP90s), blue stars represent soybean HSP90s (GmHSP90s), green circles represent Arabidopsis HSP90s (AtHSP90s), blue squares represent quinoa HSP90s (CqHSP90s), purple check marks represent maize HSP90s (ZmHSP90s).

To further reveal the evolutionary relationship of the *HSP90* genes, we performed a phylogenetic analysis of 49 HSP90 protein sequences including 11 in quinoa, 11 in maize (*Zea mays*), 8 in rice (*Oryza sativa*), 12 in soybean (*Glycine max*), and 7 in *Arabidopsis thaliana*, (Table S2). As shown in Figure 1, the phylogenetic tree showed that quinoa genes were more closely related to those in *Arabidopsis thaliana* and soybean, which may be related to the fact that they were dicotyledonous plants and were closer to the same ancestors in the evolution of species. According to the phylogenetic tree, they were divided into three subgroups or classes, including 17 members in class 1, 8 members in class 2, and 24 members in class 3. CqHSP90 has six members in class 3, four members in class 1, and two members in class 2.

### 2.3. Motif and Genetic Structure Analysis of the HSP90 Gene Family

**Figure 2:**
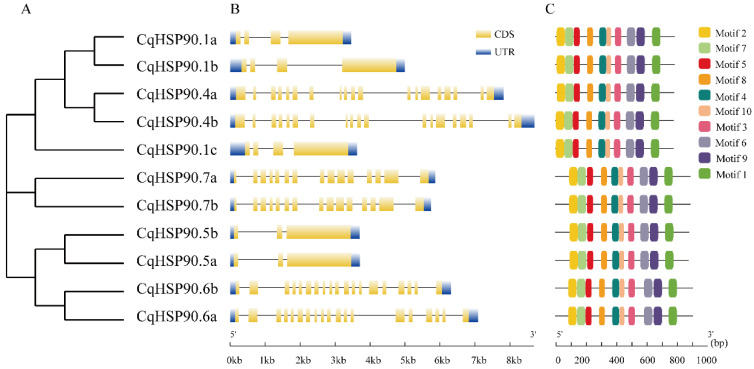
Phylogenetic relationships, conserved protein Motifs, and genetic structures of *CqHSP90*. (**A**) Phylogenetic tree was constructed with the NJ method using MEGA-7. (**B**) Structures of 11 putative *CqHSP90* genes. Orange boxes represent CDs, blue boxes represent UTRs, and black lines represent introns. (**C**) Motif distribution of CqHSP90 protein. Motifs 1–10 are represented by different colors.

To resolve the Motif composition and arrangement of *CqHSP90s*, we submitted 11 protein sequences to the MEME website and obtained a total of 10 predicted Motifs (Supplemental Table S3). Figure 2 showed that all the sequences contain these 10 Motifs, and the order of these Motifs is completely consistent, which also shows that the 10 Motifs are all with conserved sequences. In order to understand the variability of the genetic structure of the *CqHSP90* gene family in the evolution process, we carried out a comprehensive analysis of the exon–intron arrangement. Figure 2 also shows that all the 11 members have intron structures and the genetic structure between members on the same branch are extremely similar or identical. Meanwhile, there are significant differences in the number and length of introns among *CqHSP90* members. The quinoa *HSP90* genes have 2–19 introns, with *CqHSP90.6a* and *CqHSP90.6b* having the most extensive introns, and *CqHSP90.5a* and *CqHSP90.5b* having the least number of introns.

### 2.4. Analysis of Cis-Acting Elements of the CqHSP90 Gene Family

**Figure 3:**
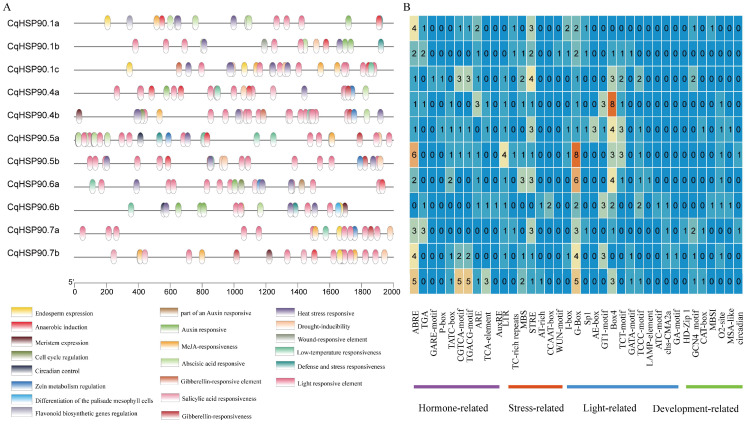
Distribution and number of cis-elements in the putative promoter of the *HSP90* genes in quinoa. Analysis of cis-elements in the promoter region of the *CqHSP90* genes. (**A**) Colored blocks represent different types of cis-elements and their positions in each *CqHSP90* genes. (**B**) Different colors and numbers indicate the number of different promoter elements in the *CqHSP90* genes.

As shown in Figure 3, the cis-acting elements in the *CqHSP90* gene promoters can be divided into four categories: plant development, stress response, hormone response, and light response. Here, eight species respond to plant development, four species respond to stress, six species respond to hormone, and 13 species respond to light (Table S4).

We found that there were as many as 13 groups of light response elements, among which G-Box (light response and binding to other pressure regulatory elements) was the most abundant in the *CqHSP90* gene family. Under low-temperature stress, *CqHSP90.5a* contained the most LTR, but *CqHSP90.1c*, *CqHSP90.1b*, and *CqHSP90.7b* did not. Among the stress-responsive elements, only one WUN-Motif was found in *CqHSP90.1b*. There were seven GCN4_Motifs (endosperm-specific expression elements) and only one HD-Zip_1 (differentiation of palisade mesophyll cells) in *CqHSP90.6b*. There were no growth and developmental elements present in *CqHSP90.1b*.

### 2.5. Heat Stress and Tissue Expression Pattern Analysis of HSP90

**Figure 4:**
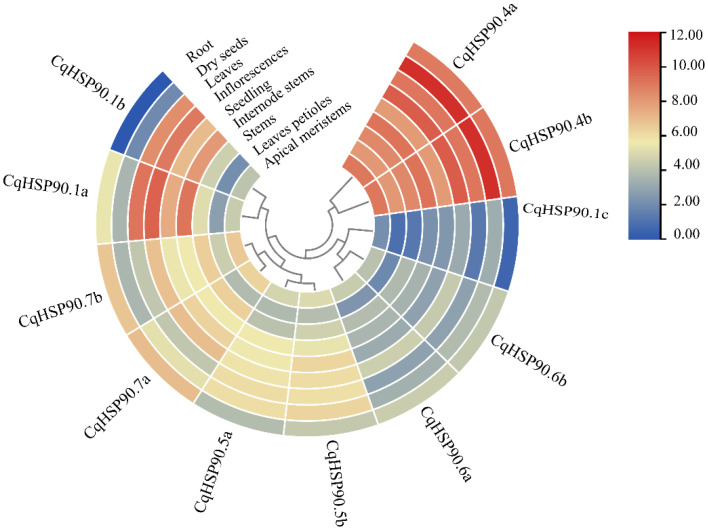
Expression pattern of *CqHSP90* in nine tissues of quinoa. Heat map showing expression levels of *CqHSP90* in nine tissues of quinoa, including apical meristems, leaves petioles, stems, internode stems, seedling, inflorescences, leaves, dry seeds, and root.

As shown in Figure 4, the expression patterns of *CqHSP90.1a* and *CqHSP90.1b* were similar in different tissues, but the expression pattern of *CqHSP90.1c* was different from the other two, and the tissue expression level of *CqHSP90.1c* was low during development. The expression levels of *CqHSP90.1c* and *CqHSP90.1b* were similar except in roots. The expression levels of *CqHSP90.4a* and *CqHSP90.4b* were relatively high during the whole development process, and the expression level was the highest in the seeds. The expression patterns of other homologous genes in different tissues were similar, and the expression levels were not high.

**Figure 5:**
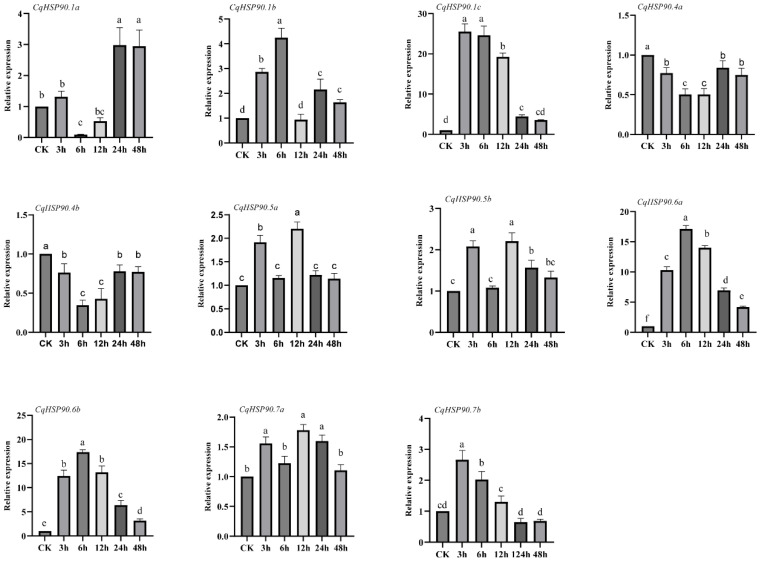
Expression pattern analysis of *CqHSP90* genes under heat stress. The mean expression levels and standard deviations were calculated using data from three biological replicates. The vertical bars indicate the standard deviation. The time points were 0 (CK), 3, 6, 12, 24, and 48 h, respectively. Values were mean relative expression ± S.D. (*n* = 3). Different lowercase letters indicate significance of differences in stress treatments at different time points (*p* < 0.05).

In order to investigate the expression changes of *CqHSP90* genes in quinoa under heat stress, we used qRT-PCR to detect the expression levels of 11 *CqHSP90* genes. As shown in Figure 5, *CqHSP90.1a* was significantly down-regulated (0.09 times) at 6 h compared with 0 h, and significantly up-regulated at 24 h compared with 0 h, and reached the maximum (3 times); *CqHSP90.1b* showed an upward trend in the early stage, and significantly up-regulated (4 times) at 6 h compared with 0 h, showing an overall upward trend; the expression of *CqHSP90.1c* was significantly up-regulated at 3 h (25.5 times), 6 h (24.6 times), and 12 h (19.3 times) compared with 0 h. At 24 h and 48 h, it also showed significant up-regulation (4.4 and 3.5 times, respectively), which was the highest expression member in all stress times, and the overall trend was upward. *CqHSP90.4a* and *CqHSP90.4b* showed a similar down-regulation trend at different time points, and both *CqHSP90.4a* and *CqHSP90.4b* showed a significant down-regulation trend at 6 h compared with 0 h (0.44 and 0.34 times, respectively). The expressions of *CqHSP90.5a* and *CqHSP90.5b* were similar at different time points and showed an overall upward trend. *CqHSP90.5a* was significantly up-regulated to a maximum (2.3 times) at 12 h compared to 0 h, while *CqHSP90.5b* was significantly up-regulated to a maximum (2 times) at 3 h compared to 0 h. The expression of *CqHSP90.6a* and *CqHSP90.6b* showed a similar trend at different time points, reaching the peak at 6 h, significantly up-regulated compared with 0 h (17.5 and 16.9 times, respectively), and down-regulated to the minimum at 48 h. *CqHSP90.6a* was up-regulated to the maximum (17 times) at 6 h compared with 0 h, and its expression level was second only to *CqHSP90.1c*. *CqHSP90.7a* reached a maximum (1.78 times) at 3 h compared with 0 h, and then decreased to a minimum (1.1 times) at 48 h. *CqHSP90.7b* was up-regulated to the maximum value (2.67 times) at 12 h compared with 0 h, and then gradually down-regulated to the minimum value (0.65 times) at 24 h compared with 0 h. Therefore, we selected *CqHSP90.1c* and *CqHSP90.6a* for subsequent studies. 

### 2.6. Protein 3D Structure Prediction

**Figure 6:**
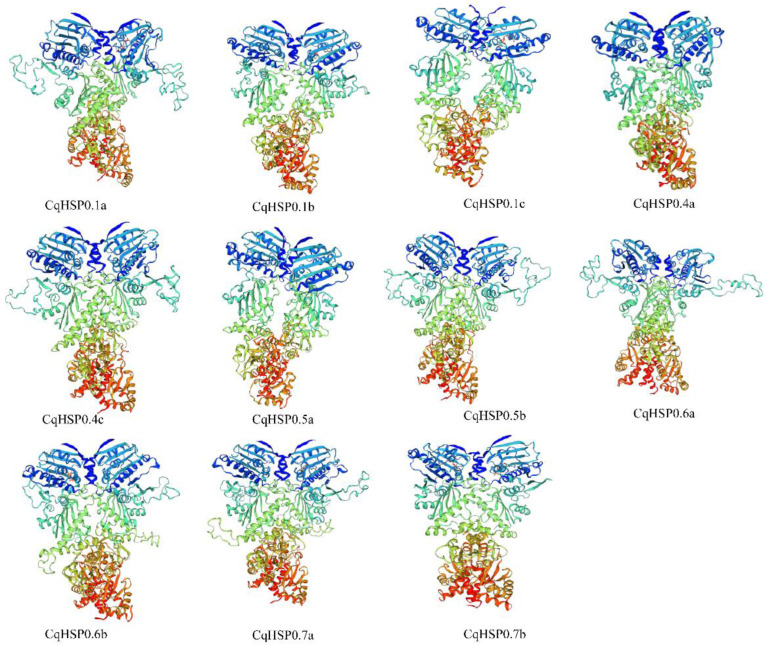
CqHSP90 three-dimensional (3D) protein structure. The broad bars are beta sheets, the helices are alpha helices, and the thin loops are coils.

To obtain information on the three-dimensional structure of the CqHSP90 protein, we used the SWISS-Model website for protein homology modeling. The gene family member proteins shared 41–67% homology with the corresponding proteins as templates (Supplemental Table S6). The three-dimensional structure of the protein constructed using the same homologous template 6xlc.1a is shown in Figure 6. The structure of CqHSP90 family proteins is basically similar, apart from CqHSP90.6a, which is slightly different from the others. The global model mass estimate (GMQE) ranges from 0.58 to 0.77, and the QMEAN z-score is between −1.76 and −3.71, indicating that the geometry of the predicted three-dimensional structure is satisfactory. All CqHSP90 proteins have β-turns, which play an important role in protein phosphorylation, glycosylation, and hydroxylation.

### 2.7. Analysis of Protein–Protein Interaction Network

In order to further investigate the interaction between CqHSP90 and its partners, the interaction network of homologous *Arabidopsis thaliana* model proteins was analyzed through the STRING website. All 11 CqHSP90 proteins were homologous to *Arabidopsis thaliana* ones, with sequence similarities ranging from 71.3 to 93.9% (Table S7).

**Figure 7:**
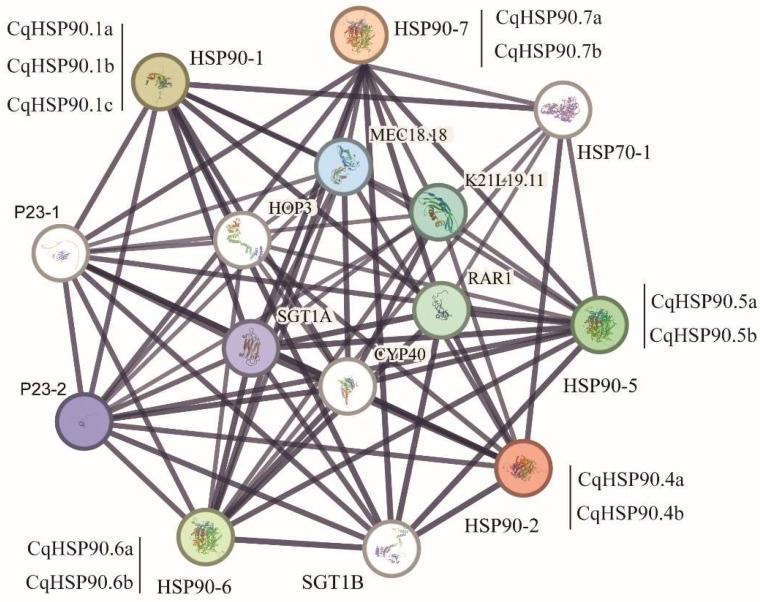
CqHSP90 protein interaction network analysis based on Arabidopsis homology. Nodes indicate lines of experimentally determined interactions to connect, line thickness indicates strength of data support, framework proteins are functional partners that interact with HSP90, and unconnected HSP90 has no known relationship, using a high confidence level.

As shown in Figure 7, CqHSP90 proteins not only interact with each other, but also are highly linked to other proteins, such as heat shock protein 70 (HSP70), suppressor of the G2 allele of skp (SGT), RAR, CytochromeP (CYP), and heat shock protein (HSP)70-HSP90 organizing protein (HOP), suggesting that they may participate in some biological processes together (Table S8). It may cooperate with different types of partners to cope with the pressure of different environmental choices, although its mechanism needs further study.

### 2.8. Subcellular Localization and Acquisition of Transgenic Material

**Figure 8:**
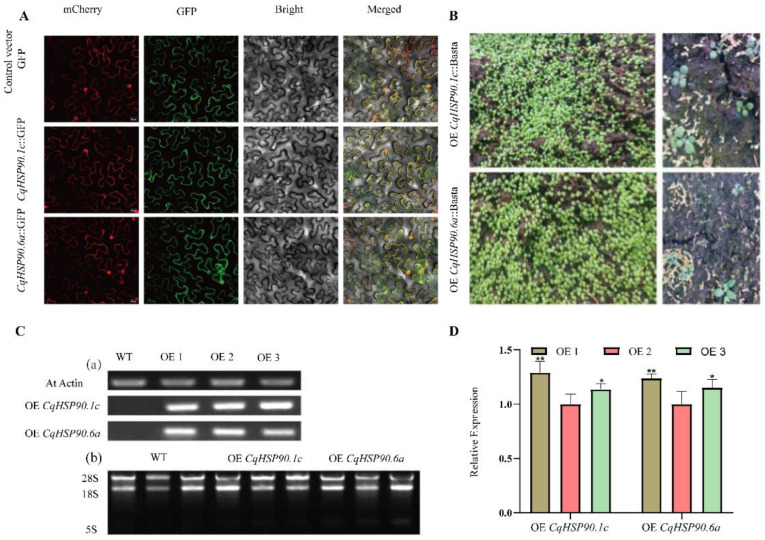
Subcellular structure localization and acquisition of overexpression positive homozygous line. (**A**) Subcellular localization of CqHSP90.1c and CqHSP90.6a proteins in tobacco leaf epidermal cells. Scale bar = 20μm. (**B**) Positive line screening using Basta for an overexpression vector constructed with *CqHSP90.1c* and *CqHSP90.6a* genes. (**C**) DNA molecular level verification of transgenic positive lines (a), and RNA extraction of wild-type and transgenic positive lines (b). (**D**) qRT-PCR results of lines homozygous for overexpression of different genes. The data shown are the mean (± SD) of three replicates (* *p* < 0.05, ** *p* < 0.01).

To analyze the intracellular location of CqHSP90.1c and CqHSP90.6a proteins, we constructed recombinant plasmids to express the cDNAs of CqHSP90.1c and CqHSP90.6a fused to the N-terminus of the GFP reporter gene (Figure S2). This construct was placed under the control of the CaMV 35S promoter and then co-localized with the ER membrane Maker of *N. benthamiana*. Figure 8A showed that both CqHSP90.1c and CqHSP90.6a are located on the endoplasmic reticulum membrane and are membrane-localized proteins. Meanwhile, in order to further investigate the role of CqHSP90.1c and CqHSP90.6a under high-temperature tolerance, we constructed an overexpression vector (Figure S2), driven by 2 × 35S strong promoter, and overexpressed CqHSP90.1c and CqHSP90.6a in *Arabidopsis thaliana* using *Agrobacterium*-mediated transformation. Under Basta screening, the untransformed negative *Arabidopsis thaliana* plants were albino and died, while the positive seedlings remained green and healthy (Figure 8B). As shown in Figure 8C, the results of DNA molecular level detection for the screened transgenic lines showed that *AtActin* could be amplified in all samples, while *CqHSP90.1c* and *HSP90.6a* could only be amplified in their corresponding transgenic lines (a). RNA was then extracted from the molecularly detected transgenic lines for qRT-PCR detection (b). At the same time, we detected the transcription level of homozygous transgenic lines (OE1, OE2, and OE3) of different genes. The lowest transcription level of *CqHSP90.1c* in OE2 was 1, the transcription level in OE1 was 1.3 times higher than that in OE2, and the transcription level in OE3 was 1.1 times higher than that in OE2; The lowest transcription level of *CqHSP90.6a* was 1 in OE2, which was 1.2 times higher in OE1 and 1.1 times higher in OE3.

### 2.9. Overexpression of CqHSP90 Enhances High-Temperature Tolerance in Arabidopsis thaliana

In order to investigate the biological role of *CqHSP90.1c* and *CqHSP90.6a* for high-temperature tolerance, 20-day-old WT and transgenic homozygous expression lines identified at the DNA level and RNA transcription level were treated at 40 °C for 3 days. Then, the photosynthetic physiological indexes of *Arabidopsis thaliana* were detected using the IMAGING PAM instrument. At the same time, samples were taken from the same parts of different plants, and RNA was extracted for qRT-PCR.

**Figure 9:**
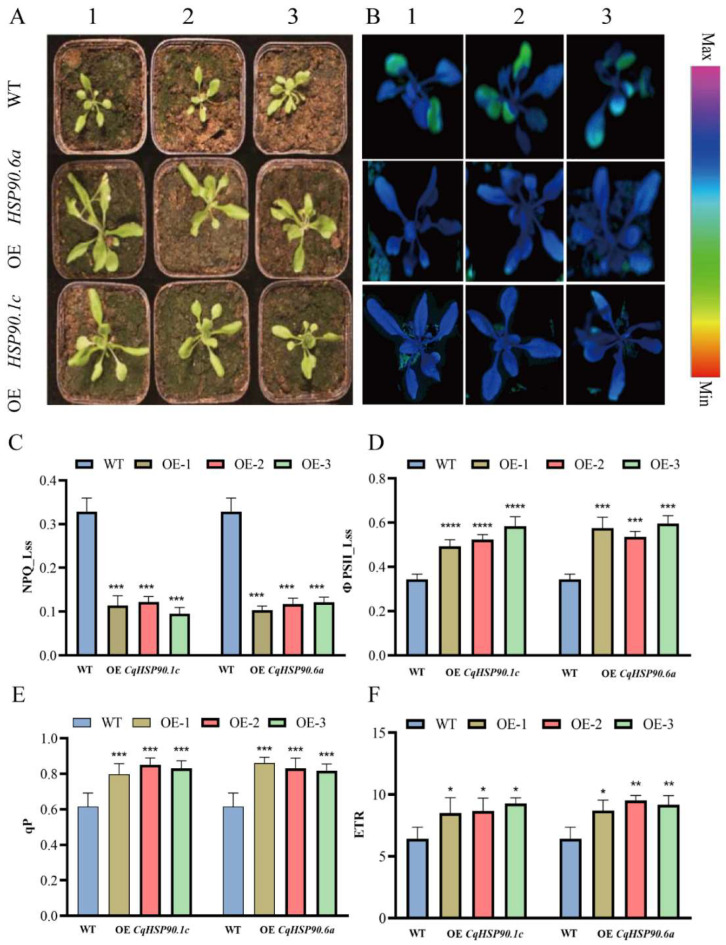
Detection of physiological indexes of wild-type and overexpression *A. thaliana* after heat treatment. (**A**) *A. thaliana* seedlings of homozygous lines overexpressing different genes and the wild type after heat treatment. (**B**) Fluorescence patterns of homozygous lines overexpressing different genes and wild-type PSII (actual photosynthetic efficiency) after heat treatment. 1, 2 and 3 in (**A**,**B**) represent representative plants of different transgenic lines. (**C**) NPQ (non-photochemical quenching coefficient) levels of wild-type and homozygous lines overexpressing different genes after heat treatment. (**D**) PSII levels of wild-type and homozygous lines overexpressing different genes after heat treatment. (**E**) Expression levels of wild-type and homozygous line overexpressing different genes after heat treatment. (**F**) ETR (electron transport efficiency) levels of wild-type and homozygous lines overexpressing different genes after heat treatment. Statistical significance was determined through one-way analysis of variance (* *p* < 0.05, ** *p* < 0.01, *** *p* < 0.001 and **** *p* < 0.0001).

As shown in Figure 9A, PSII of *CqHSP90.1c* and *CqHSP90.6a* overexpression lines after heat treatment showed significantly better tolerance to heat stress than the wild type. Transgenic *Arabidopsis thaliana* leaves were wider and thicker with larger leaf area, while WT leaves showed obvious leaf shrinkage and wrinkling. The chlorophyll fluorescence performance of YII also showed that the leaves of WT had large green areas after heat treatment, which gradually extended from the edge to the inside of the leaves, representing the declining of the actual photosynthetic capacity of the leaves. There was no significant difference between OE *CqHSP90.1c* and OE *CqHSP90.6a* in the PSII (actual photosynthetic efficiency) fluorescence performance, but there was some difference in the physical appearance. From the chlorophyll fluorescence parameter PSII (Figure 9D), we can see that after heat treatment, the overexpression lines are healthier than WT plants, which means that the overexpression plants have higher actual photosynthetic efficiency and better plant cell status. This was also confirmed by the ETR (electron transport efficiency) (Figure 9F), where the overexpressed plants showed a faster electron transport efficiency than wild-type plants, thus ensuring a higher photosynthetic efficiency. Compared with the wild type, the overexpression lines also had a higher level of NPQ (non-photochemical quenching coefficient), which was a kind of photoprotective ability of the plant to absorb light energy for heat dissipation to maintain its own health, which also confirmed from the fact that the overexpression lines had a strong heat dissipation ability to slow down the thermal damage of the plant.

## 3. Discussion

At present, extreme ecological environments of the earth occur frequently. Affected by the environment, the mRNA transcription levels of many genes in the plant body could be seriously affected, and then the synthesis of protein could be inhibited, which will have adverse effects on the body and seriously affect the survival of the plant [31]. In these circumstances, a class of highly conserved proteins called heat shock proteins (HSPs) are rapidly synthesized to ensure the normal transcription and translation of the body, so as to ensure the survival of the plant body in adverse environments. Among them, the *HSP90* gene family has been proved to play an important role in stress signal transduction, folding of receptors, transcription factors and kinases, and physiological processes [32,33,34], and to help plants survive adverse conditions [35]. However, as an excellent crop with strong resistance and adaptability to extreme environment, the *HSP90* gene family of quinoa has not yet been identified. In the present work, we attempted to identify the quinoa *HSP90* gene family members based on the existing omics data and bioinformatics methods.

The *CqHSP90* gene family was identified by bioinformatics methods, and 10 members were obtained. All *CqHSP90* proteins were acidic, which was consistent with the results of *Arabidopsis thaliana* and *Nicotiana tabacum* HSP90 proteins [24,36]. The subcellular localization of *CqHSP90* family members is consistent with previous *Arabidopsis thaliana* studies, mainly distributed in chloroplasts, mitochondria, nuclei, endoplasmic reticulum, and cytoplasm [24]. Promoter analysis of *CqHSP90* genes showed that *CqHSP90* promoter contained a variety of regulatory elements, such as cis-acting regulatory elements necessary for hormone response, stress response, meristem expression, photoperiod regulation, and so on, among which light-responsive elements were the most. This is consistent with the results of previous studies on the *HSP90* gene family in cucumber [10]. These may further indicate that HSP90 genes may play a key role in protein degradation, gene silencing, and protein trafficking. A heat map of *CqHSP90* tissue expression showed that *HSP90.4a* and *HSP90.4b* had high expression level in seeds; *HSP90* of *Brassica napus* also had a high expression level during seed germination, and *HSP90.1* of *Arabidopsis thaliana* was strongly expressed in embryos of mature seeds. *HSP90* plays an important role in seed development and germination [11,12]. The expression levels of *CqHSP90.4a*, *CqHSP90.4b*, *CqHSP90.7a*, and *CqHSP90.7b* were relatively higher in the stem, flower, and root meristem of normal quinoa, while the deletion of *AtHSP90.7* resulted in the disorder of stem, flower, and root meristem, the increase in floral organs, and the thickening and elongation of pollen tubes in *Arabidopsis thaliana* [37]. It was concluded that *CqHSP90.4a* and *CqHSP90.4b*, *CqCqHSP907a*, and *CqHSP907b* played an important role in the regulation of flower development and root meristem of quinoa. In order to explore the similarity and function of CqHSP90 proteins, we performed homology modeling of 11 CqHSP90 proteins and revealed the similarity rate of 41–67%. Meanwhile, we performed the analysis of PPI for CqHSP90 proteins based on the homology information of *Arabidopsis thaliana* (Table S7). The results demonstrated that, in addition to HSP90, a large number of other proteins such as HSP70, SGT, RAR, CYP, HOP, and P23 were also enriched in the network. In tomato, HSP70 and HSP90 directly interact with heat shock transcription factors (HSFs) to regulate downstream gene expression [38]. To cope with heat stress, SGT1 plays an important role in plant resistance to pathogens [39]. SGT also plays an important role in SCF-mediated ubiquitination by coupling HSP90 to the SCF complex to achieve ubiquitination of HSP90 client proteins [40]. Cofactors SGT1, HSP90, and RAR1 were observed to stabilize plant disease resistance (R) proteins in *Arabidopsis thaliana*, barley, and tobacco [41,42,43]. We suggest that quinoa may also have the potential ability to cope with biotic stress. It was demonstrated that HSP90 binds to a complex of CYP40, Argonaute 1(AGO1), and some small RNA duplexes to complete the assembly of RNA-Induced Silencing Complex (RISC) assembly in tobacco, which has important implications for post-transcriptional gene silencing [44]. Under salt stress, HOP1/2 stimulated the nuclear accumulation of HSP90-BIN2 complex, which enhanced the phosphorylation of transcription factor Brassinazole Resistant 1 (BZR1) and promoted the accumulation of Brassinosteroids (BR) to promote plant growth [45]. As a co-chaperone of HSP90, P23 stabilizes the conformational state of HSP90 through inhibiting ATP hydrolysis [46]. This provides evidence that different types of chaperones of CqHSP90 cooperate with each other under environmental stress, which is worthy of further study.

The expression patterns of *CqHSP90.1a*, *CqHSP90.1b*, and *CqHSP90.1c* in the same evolutionary branch were different, and *CqHSP90.1c* had the largest up-regulation. This is consistent with the better tolerance of *AtHSP90.7* to heat stress [47]. The others in the same evolutionary branch are similar except that *CqHSP90.7a* and *CqHSP90.7b* have some differences. In the comparative transcriptomic study of pepper, it was shown that the expression of *HSP90* in heat-resistant varieties was higher than that in heat-sensitive varieties [48]. These results indicate the tolerance of *HSP90* to heat stress. In order to further investigate the biological role of the functional sites of *CqHSP90* genes under high temperature, we cloned the full-length CDS of *CqHSP90.1c* and *CqHSP90.6a* of quinoa, and constructed the localized expression vector and overexpression vector for subcellular localization of tobacco and transgenic *Arabidopsis thaliana*. We found in the KEGG website that HSP90 involves in the endoplasmic reticulum-associated degradation (ERAD) pathway, which stabilizes the intracellular environment through ubiquitinating misfolded proteins and transporting them to the proteasome to degrade misfolded proteins and unfolded peptides. This may explain why CqHSP90.1c and CqHSP90.6a are located on the endoplasmic reticulum membrane. Subcellular localization results showed that CqHSP90.1c and CqHSP90.6a were localized in the endoplasmic reticulum (ER), which indicated that the major functional site of CqHSP90.1c and CqHSP90.6a was E.R.

From the phenotypic and fluorescence analysis of heat stress in overexpressed *Arabidopsis thaliana* and wild-type *Arabidopsis thaliana*, we can see that *Arabidopsis thaliana* overexpressed and showed stronger thermotolerance than wild-type *Arabidopsis thaliana*. After heat stress treatment, the leaves of wild-type *Arabidopsis thaliana* showed partial atrophy and wrinkling, while the leaves of transgenic *Arabidopsis thaliana* overexpressing *CqHSP90* genes showed less wilting; Y (II) fluorescence images also showed that the overexpressed *Arabidopsis thaliana* had higher photosynthetic efficiency than wild-type *Arabidopsis thaliana* after heat stress treatment, and the wild-type *Arabidopsis thaliana* had low photosynthetic efficiency at the edge of leaves. HSP90-RNAi experiments on *Arabidopsis thaliana* also proved this point. Compared with the wild type, *Arabidopsis thaliana* after HSP90-RNAi showed delayed leaf development, narrow leaves, and low chlorophyll content, which affected photosynthesis [1]. More interestingly, *CqHSP90.1c*, which is highly overexpressed in *Arabidopsis thaliana*, showed a faster growth rate than *CqHSP90.6a* and *CqHSP90.6b*, and began to bolt and grow inflorescences. Results of phenotypic studies in wild-type and transgenic *Arabidopsis thaliana* after heat treatment showed that overexpression of *CqHSP90.1c* and *CqHSP90.6a* enhanced the tolerance to high temperatures of plants. Under high-temperature stress, the physiological and biochemical processes of plants are affected in different degrees. Studies have shown that plants exposed to high temperature can lead to oxidative stress and produce a large number of ROS, which can damage plant cells, affect chlorophyll content, and inhibit photosynthesis [49]. The results of this work also showed that high temperature reduced the photosynthesis efficiency of plants and affected the normal growth of plants, but the overexpression of plants maintained a better healthy state compared with the wild type. The ETR (electron transport efficiency) of OE *CqHSP90.1c* and OE *CqHSP90.6a* plants maintained at a higher level compared with WT, thus ensuring a higher PSII level. This indicates that both *CqHSP90.1c* and *CqHSP90.6a* have positive regulatory effects.

## 4. Materials and Methods

### 4.1. Genome-Wide Identification of Quinoa HSP90

Candidate family members of *HSP90* genes in quinoa were identified through Phytozome (https://phytozome-next.jgi.doe.gov/, accessed on 8 April 2024) and the whole quinoa genome data was downloaded. A protein database was built and download from the Pfam database (http://pfam.xfam.org/, accessed on 8 April 2024). The HMM files of HATPase_C (PF02518) and HSP90 domain (PF00183) were downloaded and the quinoa protein library was screened based on the HMM files (*E*-value < 1 × 10^−20^) to obtain candidate *HSP90* gene family members [50]. Meanwhile, the obtained sequences were uploaded to the NCBI CDD (https://www.ncbi.nlm.nih.gov/cdd, accessed on 8 April 2024) and SMART (https://smart.embl.de/, accessed on 8 April 2024) to remove redundant sequences and pseudogenes and to obtain the final *HSP90* gene family member sequences.

### 4.2. Subcellular Localization and Physical and Chemical Properties Analysis

The online tool ExPASy (https://web.expasy.org/protparam/, accessed on 8 April 2024) was used to estimate the theoretical isoelectric point, the number of amino acids, and the molecular weight of proteins [51]. WoLF PSORT (https://wolfpsort.hgc.jp/, accessed on 8 April 2024) was used to predict the subcellular localization of quinoa HSP90 family proteins [52].

### 4.3. Phylogenetic Analysis

The ClustalW tool of MEGA11 was used to test the data from Phytozome13 (https://phytozome-next.jgi.doe.gov/, accessed on 8 April 2024) [53], for the rice and quinoa from TAIR (https://www.arabidopsis.org/, accessed on 8 April 2024) and from NCBI (https://www.ncbi.nlm.nih.gov/, accessed on 8 April 2024) [54]. A Neighbor-Joining (NJ) phylogenetic tree was constructed using the maximum proximity method with the following system parameters: model “Poisson model”, gap setting "Pairwise deletion". The bootstrap value of the validation parameter was set to 1000 [55].

### 4.4. Conserved Motifs and Gene Structures of the HSP90 Gene Family 

We utilized the MEME online tool to perform conserved Motif analysis of quinoa HSP90 protein with the Motif number set to 10 and the width set to 15–50 amino acids (https://meme-suite.org/meme/, accessed on 8 April 2024) [56]. At the same time, CDS sequences and genetic sequences obtained from NCBI were uploaded to the GSDS website for genetic structure analysis of introns and exons (https://gsds.gao-lab.org/Gsds_help.php, accessed on 8 April 2024) [57].

### 4.5. Analysis of Cis-Acting Elements in the HSP90 Gene Family

In order to explore the possible stress-related cis-acting elements in the promoter sequence of *CqHSP90* genes, the upstream region (2kb) of *CqHSP90* genes were submitted to PlantCARE database for cis-acting element analysis (https://bioinformatics.psb.ugent.be/webtools/plantcare/html/, accessed on 8 April 2024) [58].

### 4.6. Protein 3D Structure Analysis and Protein–Protein Interaction Network

Website SWISS-MODEL (https://swissmodel.expasy.org/, accessed on 8 April 2024) was used to predict the three-dimensional structure of the CqHSP90 protein. Quality criteria GMQE scores were ranged from 0 to 1, with higher scores indicating more reliable models. A QMEAN score around 0 indicates high quality, and −4 or below indicates low quality. To predict the relationship between HSP90 protein and other related proteins, the HSP90 protein sequence was submitted to STRING (https://cn.string-db.org/, accessed on 8 April 2024). *Arabidopsis thaliana* was used as the reference organism. The 1st and 2nd shells were both set to 5, the minimum required interaction score was set to a high confidence level of 0.700, and the remaining settings were left as default.

### 4.7. Plant Treatment and Expression Pattern Analysis

The quinoa material used in this experiment was YT167. Using RNA-Seq data from the existing PRJNA394651 project, different tissues of quinoa were analyzed. The expression profiles of the *CqHSP90* gene family in different tissues (apical meristem, petiole, stem, internode stem, seedling, catkin, leaf, mature seed, and root) were visualized by heat mapping. Meanwhile, qRT-PCR was used to analyze the expression of *CqHSP90* gene family members under heat stress. Firstly, the quinoa seeds were sowed in small square seedling boxes and treated with abiotic stress 20 days after sowing, with seedlings showing six expanding leaves). The experimental conditions were 16/8 h (light/dark) photoperiod, 70% relative humidity, 24 °C/20 °C (day/night) temperature regime for the control group, and 40 °C constant temperature for the experimental group. The leaf samples were collected and frozen in liquid nitrogen at 0, 3, 6, 12, 24, and 48 h, respectively, and stored at −80 °C for RNA extraction. Each biological sample was taken from three different plants, and three biological replicates were set for each time point. RNA extraction was performed using RNA isolater Total RNA Extraction Reagent and cDNA synthesis was performed using HiScript II Q Select RT SuperMix for subsequent qRT-PCR analysis, calculated as 2^−∆∆ct^ [59]. The reagents used were purchased from Novizan Biotechnology Co., Ltd. (Nanjing, China). Quantitative PCR was performed using LightCycler 480 system to determine the gene expression level, and the quinoa actin *(CqActin*) gene was used as the internal standard gene.

### 4.8. Subcellular Localization of CqHSP90.1c and HSP90.6a

The full-length cDNAs of *CqHSP90.1c* and *CqHSP90.6a* were amplified and the stop codons were removed. We double digested pCAMBIA1302 (plasmid kept in the laboratory) using FlyCutNcol and FlyCutSpeI restriction endonucleases, and then integrated the PCR product obtained in the previous step. The fusion proteins (pCAMBIA1302-*CqCqHSP90.1c-*GFP and pCAMBIA1302-*CqHSP90.6a-*GFP) were then constructed. The plasmid was introduced into *A. tumefaciens* GV3101, and mixed with *A. tumefaciens* carrying pCAMBIA1300-35S-E.R. mCherry Maker plasmid in a 1:1 ratio, and injected into tobacco leaves. Three days later, the subcellular localization was observed under AXR with NSPARCconfocal microscope (Nikon, Tokyo, Japan).

### 4.9. Plasmid Construction and Acquisition of Transgenic Plant Materials

Plant individuals with a high induction of *CqHSP90.1c* and *CqHSP90.6a* after heat treatment were selected for further analysis. *CqHSP90.1c* and *CqHSP90.6a* were amplified using full-length cDNA PCR for vector construction. The PCR products obtained in the previous step were integrated with the plasmid pSCZ3 and subjected to double digestion and linearization using FlyCut *Bam*HI and FlyCut *Xba*I restriction endonucleases. Fusion expression proteins (pSCZ3 Bar-*CqHSP90.1c* and pSCZ3 Bar-*CqHSP90.6a*) were then constructed to generate overexpression vectors. The *CqHSP90.1c* and *CqHSP90.6a* genes were introduced into *Arabidopsis thaliana* by *Agrobacterium*-mediated transformation. The heterozygote seeds of T1 generation were collected with mixing samples. The seeds of T2 generation were screened using Basta at the seedling age of fully expended cotyledons. The seedlings were sprayed with 2–3-day intervals. The positive seedlings were screened and transplanted after three times of continuous spraying. The concentration used by Basta is 7.5 mg/L; its main active ingredient is phosphinothricin. The mature seeds of T2 generation were harvested and the seedlings of T3 generation were continuously screened. Phenotype analysis was carried out with T4 homozygous plants.

### 4.10. DNA Molecular Level Verification and Quantitative Expression Analysis of Transgenic Lines

To verify the normal transformation of *Arabidopsis thaliana*, we studied the expression of 2 genes in 3 independent biological replicates of wild-type and overexpressing lines (Table S5). First, we extracted DNA from transgenic plants by FastPure Plant DNA Isolation Mini Kit (Vazyme, Nanjing, China), and amplified *CqHSP90.1c* and *CqHSP90.6a* by T100 PCR instrument (Bio-Rad, Hercules, CA, USA). RNA is extracted from the identified transgenic strains using an RNA isolater Total RNA Extraction Reagent. After cDNA synthesis by HiScript II Q Select RT SuperMix, qRT-PCR analysis was performed on homozygous strains by LightCycler 480 fluorescence quantitative instrument (F. Hoffmann-La Roche Ltd., Basel, Switzerland). The relative expression was calculated by the delta–delta Ct method and normalized to the individual overexpression line with the lowest transcript abundance [60].

### 4.11. Determination of Physiological Indexes of Wild-Type and Transgenic Arabidopsis

To examine the molecular functions of *CqHSP90.1c* and *CqHSP90.6a*, we compared transgenic *Arabidopsis thaliana* with WT. Before treatment, *Arabidopsis thaliana* was selected and transplanted into a small box and cultured in a controlled incubator at 23 °C and a 8/16 H photoperiod. The 3-week-old transgenic lines and WT plants were treated at 40 °C for 3 days. Phenotypes were photographed and photosynthetic indexes were detected using IMAGING PAM with 2.2.0.0 Imaging Win (WALZ, Nuremberg, Germany).

## 5. Conclusions

In this work, 11 members of the *HSP90* gene family were identified in quinoa and were named according to their evolutionary relationship with *Arabidopsis thaliana*. Their physical and chemical properties, phylogenetic relationships, genetic structure and proteins Motif, cis-acting elements, and expression patterns were fully analyzed. *CqHSP90.1c* and *CqHSP90.6a* were significantly up-regulated under heat stress, with expression levels in homozygous OE plants increased by more than seven folds compared with those of the wild type. After heat treatment, the homozygous plants overexpressing *CqHSP90.1c* and *CqHSP90.6a* had a larger leaf area, normal leaf edge, and higher PSII level, while the WT plants grew slowly with leaf shrinkage and significantly decreased chlorosis content and PSII system. In summary, the heterologous functional verification of *Arabidopsis thaliana* overexpression of *CqHSP90.1c* and *CqHSP90.6a* indicated that HSP90 played a positive regulatory role in quinoa response to heat stress.

## Figures and Tables

**Table 1:** Physicochemical properties of the CqHSP90 gene family in *Chenopodium quinoa*.

Gene ID	Gene Name	Number of Amino Acids	Molecular Mass (kDa)	pI	Subcellular Location	Instability Index	Aliphatic Index	GRAVY
LOC110729587	*CqHSP90.1a*	703	80.9	5	Nucl	42.65	82.11	−0.62
LOC110722595	*CqHSP90.1b*	703	80.9	5	Cyto	41.65	81.96	−0.631
LOC110693874	*CqHSP90.1c*	700	81.1	5.07	E.R.	42.42	84.64	−0.629
LOC110686052	*CqHSP90.4a*	697	79.9	4.99	Cyto	37.5	83.07	−0.586
LOC110721231	*CqHSP90.4b*	697	79.9	4.99	Cyto	37.38	83.34	−0.586
LOC110711021	*CqHSP90.5a*	795	90.2	4.95	Chlo	41.44	78.35	−0.554
LOC110707029	*CqHSP90.5b*	795	90.4	4.93	Chlo	44.03	78.35	−0.562
LOC110691828	*CqHSP90.6a*	788	88.9	5.3	E.R.	40.8	82.04	−0.532
LOC110701871	*CqHSP90.6b*	784	88.4	5.24	Mito	41	82.69	−0.508
LOC110721133	*CqHSP90.7a*	810	93.1	4.83	E.R.	37.01	79.31	−0.753
LOC110720567	*CqHSP90.7b*	810	93.1	4.83	E.R.	37.43	79.19	−0.758

## Data Availability

The data presented in this study are available in the article and Supplementary Materials.

## Supplementary Materials

The following supporting information can be downloaded at https://www.mdpi.com/article/10.3390/plants14172770/s1; Figure S1. Multiple alignment of HATPase_C and HSP90 domains from CqHSP90; Figure S2. Schematic diagram of construction of different vectors; Figure S3. GO classification of BP, CC, and MF based on similarity annotation to *Arabidopsis*; Table S1. Conserved domains of HSP90 proteins in *Chenopodium quinoa*; Table S2. Information of *HSP90* genes from *Chenopodium quinoa*, *Zea mays*, *Oryza sativa*, *Glycine max*, and *Arabidopsis thaliana*; Table S3. Analysis of the 10 conserved motifs of CqHSP90 proteins in *Chenopodium quinoa*; Table S4. Analysis of cis-acting element of CqHSP90 proteins; Table S5. Primer information used in this work; Table S6. The detailed information of the predicted three-dimensional (3D) proteins; Table S7. The predicted *Chenopodium quinoa* orthologs of *CqHSP90s* with STRING; Table S8. String interactions.
