# Supplementary material for: Identification of the Chenopodium quinoa HSP90 Gene Family and Functional Analysis of CqHSP90.1c and CqHSP90.6a Under High-Temperature Stress in Transgenic Arabidopsis thaliana
Source: Plants (Basel). 2025 Sep 4;14(17):2770. doi: 10.3390/plants14172770 (PMC12430413; doi:10.3390/plants14172770)
Supplement: Supplementary file 1 [file plants-14-02770-s001.zip › Supplementary figure.pdf]

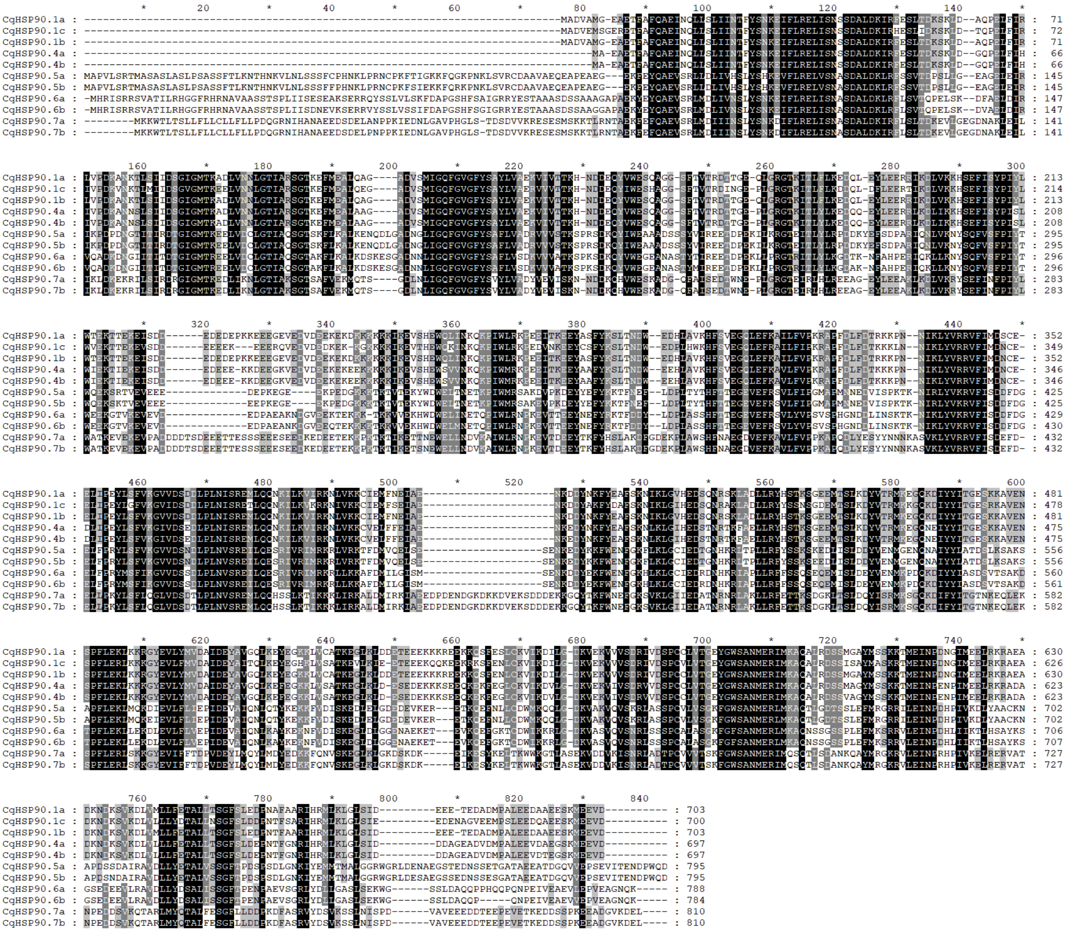

Figure S1. Multiple alignment of HATPase_C and HSP90 domains from CqHSP90. Conserved proteins are indicated by *, red for the HATPase_C domain and purple for the HSP90 domain.

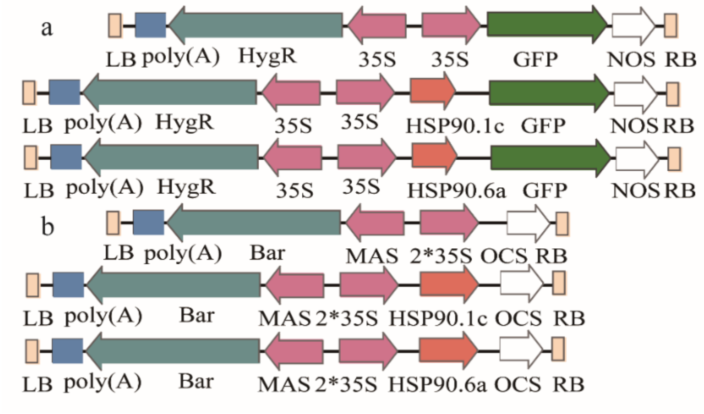

Figure S2. Schematic diagram of construction of different vectors. a: Subcellular localization backbone vector and the pCAMBIA1302-*CqHSP90*: :GFP new construction vector; b: Overexpression backbone vector and the pSCZ3 Bar-*CqHSP90* new construction vector.

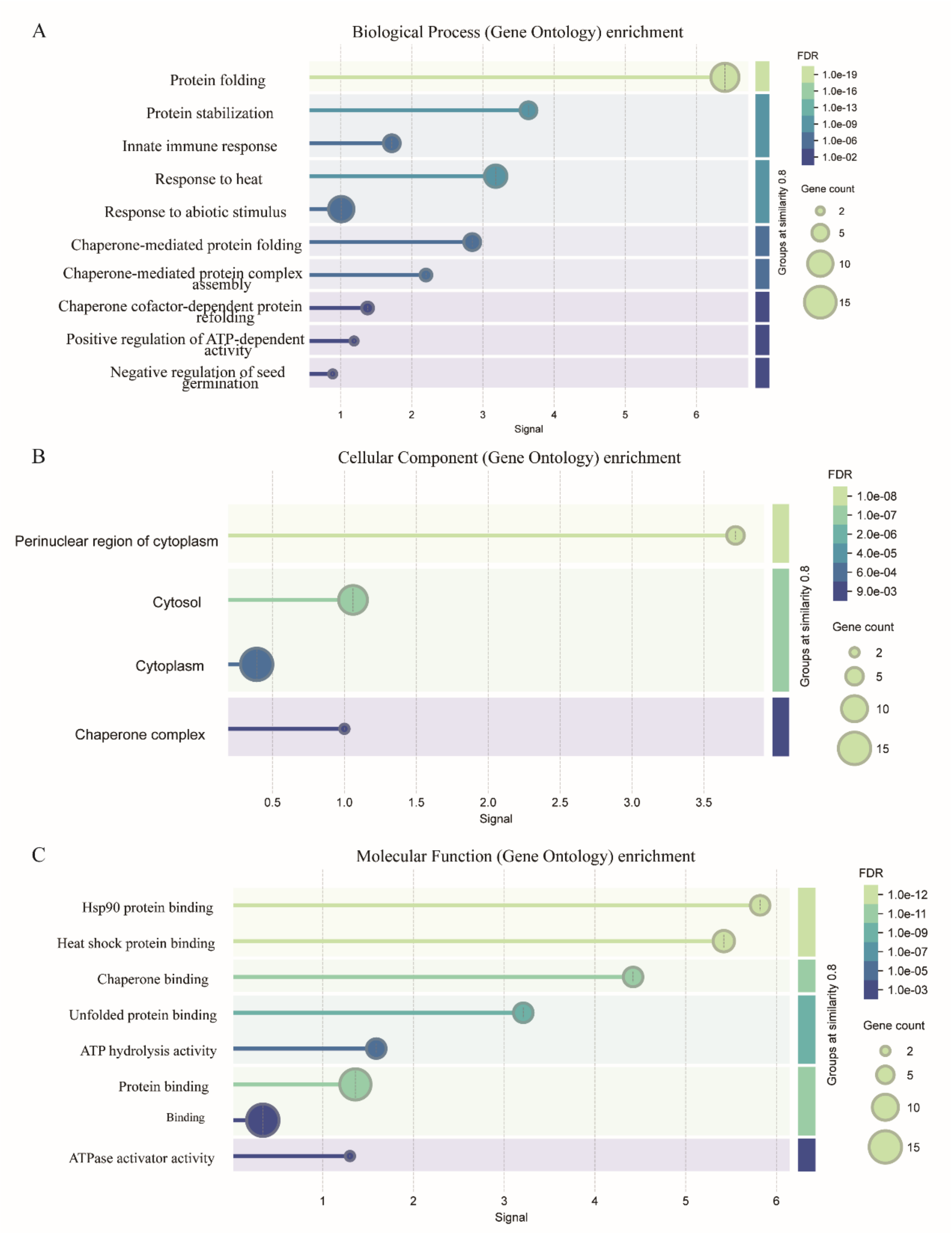

Figure S3. GO classification of BP, CC, and MF based on similarity annotation to *Arabidopsis thaliana*.
